# A Novel Change Detection Method for Natural Disaster Detection and Segmentation from Video Sequence

**DOI:** 10.3390/s20185076

**Published:** 2020-09-07

**Authors:** Huijiao Qiao, Xue Wan, Youchuan Wan, Shengyang Li, Wanfeng Zhang

**Affiliations:** 1 School of Remote Sensing and Information Engineering, Wuhan University, Wuhan 430079, China; qiaohj@whu.edu.cn; 2 Technology and Engineering Center for Space Utilization, Chinese Academy of Science, Beijing 100094, China; wanxue@csu.ac.cn (X.W.); shyli@csu.ac.cn (S.L.); wfzhang@csu.ac.cn (W.Z.); 3 Key Laboratory of Space Utilization, Chinese Academy of Sciences, Beijing 100094, China

**Keywords:** change detection, natural disasters, deep learning, threshold selection, optical flow estimation

## Abstract

Change detection (CD) is critical for natural disaster detection, monitoring and evaluation. Video satellites, new types of satellites being launched recently, are able to record the motion change during natural disasters. This raises a new problem for traditional CD methods, as they can only detect areas with highly changed radiometric and geometric information. Optical flow-based methods are able to detect the pixel-based motion tracking at fast speed; however, they are difficult to determine an optimal threshold for separating the changed from the unchanged part for CD problems. To overcome the above problems, this paper proposed a novel automatic change detection framework: OFATS (optical flow-based adaptive thresholding segmentation). Combining the characteristics of optical flow data, a new objective function based on the ratio of maximum between-class variance and minimum within-class variance has been constructed and two key steps are motion detection based on optical flow estimation using deep learning (DL) method and changed area segmentation based on an adaptive threshold selection. Experiments are carried out using two groups of video sequences, which demonstrated that the proposed method is able to achieve high accuracy with F1 value of 0.98 and 0.94, respectively.

## 1. Introduction

Natural disasters, such as earthquakes, tsunamis, floods and landslides, have shown a dramatically and globally increasing trend, both in frequency and intensity [1,2,3]. Accurate determination of changes on ground features associated with destructive disaster events is crucial to quick disaster response, post-disaster reconstruction and financial planning [4]. Change detection (CD) using remote sensing data can effectively capture changes before and after disasters [5,6,7], which has been widely used in various fields of natural disasters such as flood monitoring [8], landslide displacement tracking [9,10] and earthquake damage assessment [11,12], as well as relief priority mapping [13,14]. 

With the continuing growth of earth observation techniques and computer technology, massive amounts of remote sensing data for natural disaster with different spectral-spatial-temporal resolution are available for surveying and assessing changes in natural disaster, which greatly promotes the development of change detection methodologies. Many change detection approaches for natural disaster scenes have been proposed and they can be broadly divided into traditional and deep learning (DL)-based [15]. For traditional CD methods, the simplest approaches are algebra-based methods. Hall and Hay [16] firstly segmented two panchromatic SPOT data observed at different times and then detected changes through an image differencing method. Matsuoka et al. [17], on the basis of the difference between the backscattering coefficient and correlation coefficient achieved in an earthquake, applied supervised classification of the pre- and post-event optical images to present the distribution of damaged areas in Bam. These directly algebraic operations were easy to be implemented but always generated noisy outputs, such as isolated pixels or holes in the changed objects; thus, some transformation and models were used in CD researches. Sharma et al. [18] finished a damage assessment of landslides in a minimum time by pseudo color transformation and extracting the landslide affected area based on the pre- and post-earthquake Landsat-8 images. Lee et al. [19] first proposed an optimization algorithm based on Stepwise Weight Assessment Ratio Analysis (SWARA) model and geographic information system to assess seismic vulnerability. In order to overcome the limitation of the sole band and improve the identification of change detection, fusing datasets acquired from various remote sensors and geographical data are paramount to monitoring the environmental impacts of natural disasters. ElGharbawi et al. [20] estimated the crustal deformation affected by the 2011 Tohoku earthquake combined with two deformation patterns using Synthetic Aperture Radar (SAR) and GPS data. With the purpose of determining the changed buildings in forested areas, Du et al. [21] adopted the graph cuts method taking account of spatial relationships and took grey-scale similarity from old aerial images and height difference based on Digital Surface Model (DSM) generated from LiDAR data as two change detection indexes to optimize building detection. 

Due to the rapid development of computer technology, the research of traditional change detection approaches has tuned into integrating deep learning techniques in recent years. Deep learning-based methods have presented promising potentials based on the extraction of high-level features. Saha et al. [22] detected collapsed buildings from SAR images and Ji et al. [23] further put them into a random forest classifier to detect post-seismic destroyed buildings using pre- and post-disaster remote sensing images. In order to achieve higher accuracy, some new neural networks have been introduced into disaster monitoring researches. Ci et al. [24] proposed a novel Convolutional Neural Network (CNN) model in combination with a CNN feature extractor, a new loss function and an ordinal regression classifier to evaluate the degree of building damage caused by earthquakes using aerial imagery. Peng et al. [25] utilized an end-to-end CD method named UNet++ to fuse multiple feature maps from different semantic segmentation levels to generate a final change map with high accuracy. Yavariabdi et al. [26] proposed a new change detection method based on multiobjective evolutionary algorithm (MOEA), which is robust to multispectral Landsat images with atmospheric changes. In this method, the similarity index measure (SSIM) is used to generate the difference image. After that, MOEA is applied to obtain a set of multiple binary change masks by iteratively minimizing two objective functions for changed and unchanged regions and the final binary mask is optimally fused by MRF. With the purpose of improving efficiency, in Ghaffarian et al. [27], extended U-net based on deep residual (ResUnet) followed a Conditional Random Field (CRF) implementation was proposed to update the post-disaster buildings from very high resolution imagery. Alizadeh et al. [12] established a new hybrid framework of Analytic Network Process (ANP) and Artificial Neural Network (ANN) models for earthquake vulnerability assessment. To avoid labeling a massive number of data for the training network, transfer learning has received increased attention. Pi et al. [28] employed transfer learning to train eight CNN models based on You-Only-Look-Once (YOLO), so as to recognize undamaged building roofs in disaster-affected areas. Transferring learning was used by Kung et al. [29] to manage disaster by combination of data augmentation, reference model and augmented model. Li et al. [30] proposed SDPCANet by combining PCANet and saliency detection to make change detection based on SAR images, which effectively reduced the number of training samples but kept higher change detection performance.

Recently, the development of commercial video satellites and the spread of mobile devices makes it possible to thoroughly monitor the changing process in natural disaster. For example, high resolution video sequences from video satellites, such as SkySat and Jilin_1, can provide valuable data during different disaster phases [31,32]. Thus, change detection can now move from pre- and post-image analysis to almost real-time disaster monitoring using video sequences. Although change detection for natural disaster has been researched for years in the society of remote sensing, the main studies are focused on pre- and post-disaster satellite imagery, while the change detection based on video satellite for natural disaster monitoring has rarely been studied. These video sequences, which capture disaster motion change, bring a challenge for existing CD methods because the color and texture of objects usually remain the same while only the positions have been changed. 

The aim of this paper is to explore an effective method that detects the motion change in disaster from video sequences. Optical flow in the field of computer vision, is very likely to detect the pixel in this video sequence owing to its fast speed and pixel-based motion tracking. However, to fuse the result of optical flow into the final change detection map is a challenge. The generation of change detection map required the empirical threshold for optical flow, which may vary from case to case. Thus, this paper first presents the investigation of the motion detection property of the optical flow estimation algorithm based on deep learning and then proposes a novel change detection framework, OFATS, based on a new objective function for video sequence in natural disaster which combines the optical flow results and an adaptive thresholding segmentation algorithm based on the ratio of maximum between-class variance and minimum within-class variance. 

The rest of this paper is organized as follows: Section 2 briefly reviews the optical flow estimation methods. The proposed change detection method is then introduced in Section 3. In Section 4, the effective of the proposed method is tested and compared with some most commonly used CD methods using two different natural disaster datasets. Finally, the paper is concluded in Section 5.

## 2. Optical Flow Estimation Methods

Optical flow, which represents change of the pixels’ displacement vectors between image frames, is most widely used in motion tracking [33]. For example, optical flow has been used to detect human/animal movements [34,35] and medical organ lesions [36,37], robots or vehicle navigation [38,39], measure flow motion [40], airfoil deformation and surface strain [41]. With the assumption of instantaneous pixel value invariance over a short displacement, optical flow can be separated into two categories: local computation method on the basis of Lucas–Kanade (LK) method and global computation method based on Horn and Schunck (HS) formulation [42]. LK method supposes that the adjacent pixels in a sliding window share the same motion and keep locally constant [43]. However, the size of a sliding window is difficult to be determined and further affect the final accuracy [33]. HS assumes that the velocity field varies globally smoothly, which is more fit for real scenes [44]. Horn and Schunck introduced the optical constraint equation based on the combination of velocity field and gray value to build a basic algorithm of optical flow estimation [45]. However, these traditional optical flow computation methods often provide blurred boundaries and are hard to be used in real time [46,47]. Convolutional neural networks (CNNs) have a strong ability of feature extraction and speckle noise suppressing [15,48,49], which has attracted more attention to numerous computer vision tasks.

**Figure 1:**
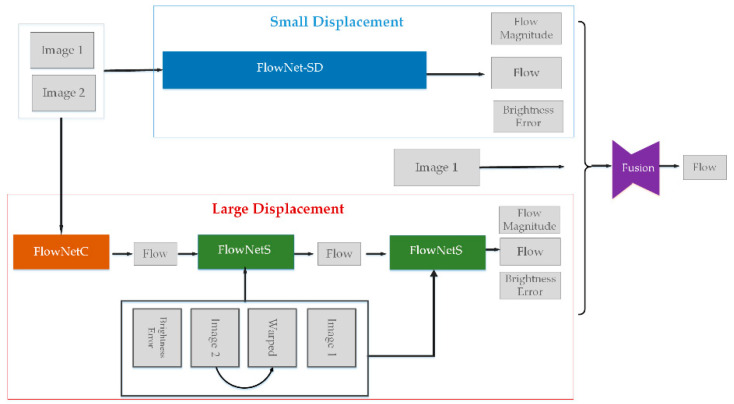
Scheme of FlowNet 2.0.

FlowNet is the first end-to-end optical flow estimation model with CNN in 2015 and it uses an encoder-decoder structure making up of convolutional and deconvolutional layer with additional crosslinks between these contracting and expanding networks [50]. For the encoder module, it is made up of nine convolutional layers and Rectified Linear Unit (ReLU), and mainly used to compute abstract features from respective fields of increasing seize, but the latter reestablishes the original resolution by an expanding upconvolutional architecture using four deconvolutional layers and ReLU active function layer. It turned out to be an achievable training network and can directly compute optical flow from two input images, but it is not competitive with fine-tuned traditional methods at accuracy and the running speed is also slower [51]. On the basis of FlowNet, FlowNet 2.0, a novel end-to-end optical flow estimation network was proposed in the winter of 2016, which can effectively solve the above-mentioned problems in close accuracy with the state-of-the-art methods while running orders of magnitude faster and be marked as a milestone for optical flow estimation based on CNN [52]. This success benefits from the following three aspects: new adding training dataset including tiny motion and real-word data, stacking numerous networks by warping operations and a novel leaning schedule of multiple datasets fusion. The schematic view of FlowNet 2.0 is shown in Figure 1. The network is separated into two parts: large displacement and small displacement optical flow network. For the computation of large displacement optical flow, two FlowNetS is combined and the warping layers are introduced as a refinement. To cope with small displacements, a smaller network, FlowNet-SD is added. Then the former stacked network and the small network are fused into FlowNet 2.0 in an optimal manner, which can achieve optimal performance on arbitrary displacements.

Optical flow estimation method based on FlowNet 2.0 has achieved considerable progress. Nevertheless, it has rarely been used to make change detection in natural disasters, as far as we know. In fact, FlowNet 2.0 based on HS generates a dense velocity field, that is, each pixel has the corresponding optical flow field [43]. In order to accurately divide pixels into changed and unchanged part based on the optical flow field, the selection of an appropriate threshold is critical. However, the threshold selection for image segmentation needs to consider the data characteristics with expert knowledge. Thus, in this paper, a novel CD framework, OFATS, for disaster detection has been proposed by combing motion detection based on FlowNet 2.0 and the adaptive threshold determination method based on a novel objective function. 

## 3. Proposed OFATS Method

**Figure 2:**
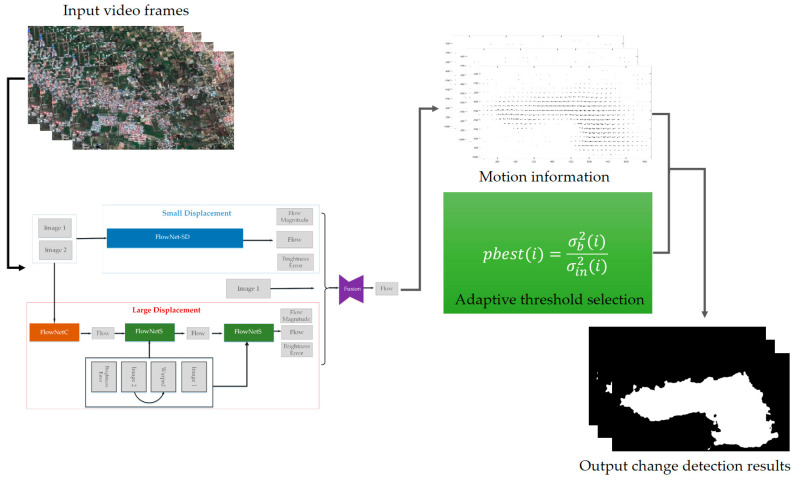
The workflow of OFATS (optical flow-based adaptive thresholding segmentation).

In this section, OFATS, the automated CD framework for natural disaster detection from video sequence is proposed and the workflow is as shown in Figure 2. 

It consists of two main steps: motion detection where FlowNet 2.0, the optical flow estimation method based on deep learning, is introduced to compute the displacement and change boundary extraction based on an adaptive threshold determination algorithm which takes the ratio of maximum between-class variance and minimum within-class variance as the new objective function. Specially, two optimization strategies are proposed: narrowing the searching range of potential thresholds and dynamic normalization of motion information.

### 3.1. Motion Detection

**Figure 3:**
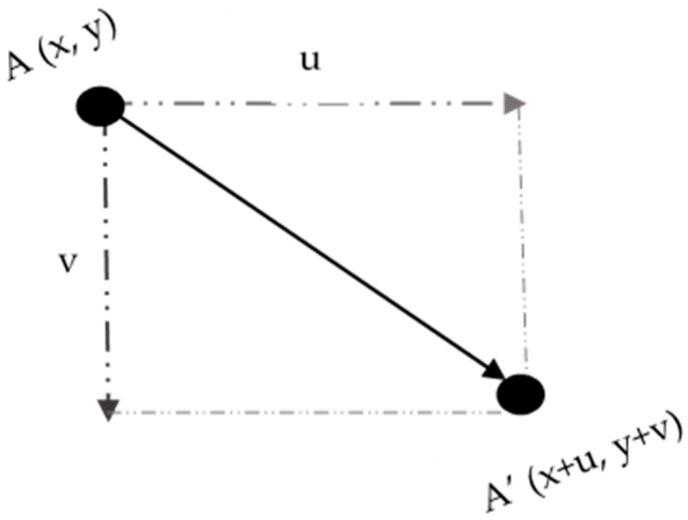
Illustration of displacements based on FlowNet 2.0.

In this paper, the pixel displacements in horizontal and vertical are calculated by FlowNet 2.0, denoted as u(x,y) and v(x,y), respectively, as shown in Figure 3.

The displacement can be calculated as follow:

(1) $$r\left(x,y\right)=\sqrt{u{\left(x,y\right)}^{2}+v{\left(x,y\right)}^{2}}$$

**Figure 4:**
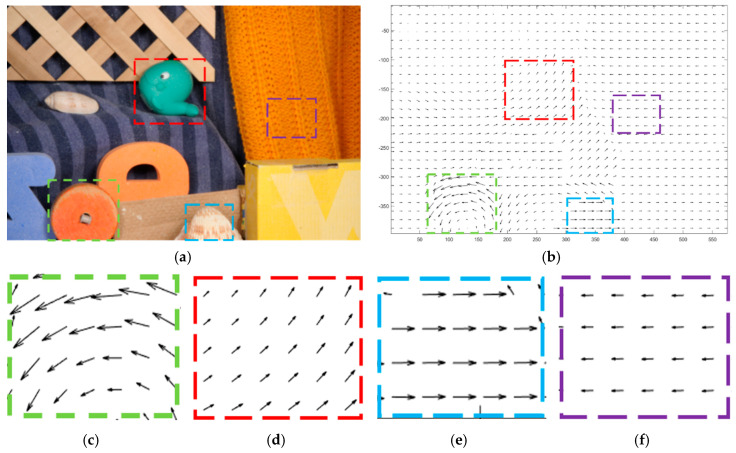
An example of displacements’ distribution (**a**) sample frame on sequence “RubberWhale” [53]; (**b**) the corresponding *r*(*x,y*) visualization based on FlowNet 2.0; (**c**–**f**) zoom-in boxes.

Figure 4 shows an example of displacements’ distribution based on sequence “RubberWhale” [53]. The sample frame and the corresponding optical flow field visualization result are shown in Figure 4a,b, respectively. In Figure 4b, the angles of arrows represent directions of each pixel’s displacement  r(x,y)  and the lengths show the magnitudes of displacements. Four boxes of different colors in Figure 4a,b representing various objects with different types of motions. In order to demonstrate the detailed difference of four boxes in Figure 4b, they are zoomed in Figure 4c–f. Overall, these figures show that the changed area can be roughly determined by the magnitudes and directions of optical flow. Given that magnitude changes are more obvious to detection, a proper segmentation threshold based on magnitude should be taken into consideration, separating the changed and unchanged part in the next step.

### 3.2. Change Boundary Extraction

After the motion detection, the next step is to determine the global optimal threshold of the displacements so as to divide the changed and unchanged part which can be viewed as a two-class classification problem. Based on displacements characteristics, the novel objective function and two optimizing strategies for the optimal threshold selection are proposed, respectively. 

#### 3.2.1. The Objective Function

For any CD algorithm, the key factor differentiating change from non-change is the objective function. The goal of setting the objective function is to find the global optimal threshold which can maximize the between-class variance and minimize the within-class variance at the same time by exploring a finite set of the possible displacement values as the possible threshold. Otsu is widely used for global thresholding selection [54], but it does not work when the target and background vary widely and two classes are very unequal [55,56]. Thus, in this paper, the objective function is set as the ratio of maximum between-class variance and minimum within-class variance:

(2) $$pbest\left(i\right)=\frac{\sigma_{b}^{2}\left(i\right)}{\sigma_{in}^{2}\left(i\right)}$$

where i is the iteration number and the value range is from 0 to Num, the number of unique value of motion detection results. pbest(i) is the fitness value of ith iteration, $\sigma_{b}^{2}\left(i\right)$ and $\sigma_{in}^{2}\left(i\right)$ are the between-class and within-class variance, and the calculations are based on Equations (3) and (4), respectively.

(3) $$\;\sigma_{b}^{2}=P\left(C_{1}\right)P\left(C_{2}\right){\left(\mu_{1}-\mu_{2}\right)}^{2}$$

(4) $$\sigma_{in}^{2}=P\left(C_{1}\right)\sigma_{1}^{2}+P\left(C_{2}\right)\sigma_{2}^{2}$$

where $P\left(C_{1}\right),P\left(C_{2}\right)$, $\mu_{1},\mu_{2}$, $\;\sigma_{1}^{2}$, $\sigma_{2}^{2}$ represent probabilities of class occurrence, class mean levels and the class variances of unchanged class $C_{1}$ and changed class $C_{2}$, respectively. They are defined as following:

(5) $$P\left(C_{1}\right)=\overset{m}{\underset{i=1}{\sum }}p_{i}$$

(6) $$P\left(C_{2}\right)=\overset{n}{\underset{i=m+1}{\sum }}p_{i}$$

(7) $$\mu_{1}=\frac{1}{P\left(C_{1}\right)}\overset{m}{\underset{i=1}{\sum }}w_{i}p_{i}$$

(8) $$\mu_{2}=\frac{1}{P\left(C_{2}\right)}\overset{n}{\underset{i=m+1}{\sum }}w_{i}p_{i}$$

(9) $$\sigma_{1}^{2}=\frac{1}{m}\overset{m}{\underset{i=1}{\sum }}{\left(w_{i}-\frac{1}{m}\overset{m}{\underset{i=1}{\sum }}w_{i}\right)}^{2}$$

(10) $$\sigma_{2}^{2}=\frac{1}{n-m}\overset{n}{\underset{i=m+1}{\sum }}{\left(w_{i}-\frac{1}{n-m}\overset{n}{\underset{i=m+1}{\sum }}w_{i}\right)}^{2}$$

The displacements can be represented in *n* levels [1, 2, …, *n*] and $C_{1}$ denotes pixels with levels [1, …, *m*], and $C_{2}$ denotes pixels with levels [*m* + 1, …, *n*]. The pixel value and the corresponding percentage at level i are denoted by $w_{i}$ and $p_{i}$. 

To concluded, $\sigma_{b}^{2}\left(i\right)$ and $\sigma_{in}^{2}\left(i\right)$ are determined by the iteration threshold value based on motion detection results and the iteration threshold $w_{i}$ corresponding to the maximal fitness value pbest(i) is considered as the optimal threshold $t_{best}$. The displacement of each pixel which is larger than the optimal threshold could be classified into the changed part, while the smaller ones are unchanged.

(11) $$g\left(x,y\right)=\{\begin{array}{c} 1,\;r\left(x,y\right)>t_{best}\\ 0,\;r\left(x,y\right)\leq t_{best} \end{array}$$

However, it requires large numbers of iteration and costs much time of too many unique values in motion detection results because the changeable range of different pixels’ displacements being really wide. Thus, the optimizing strategies are further proposed for optimal threshold selection. 

#### 3.2.2. Optimizing Strategies for Threshold Selection

According to the distribution of displacement data, we proposed two strategies to optimize the threshold selection criterion: narrowing the searching range of iterations and dynamic normalization of displacements which are greater than the currently selected threshold for each iteration.

Narrowing searching range is to efficiently reduce the scope of potential thresholds determined by the wide range of experimental data. Comparing with the change of pre- and post- disasters, the video data with 30 FPS during disasters can record the whole minor change of each frame. Thus, it should be labeled as ‘change’ when pixels with displacements are larger than 1 pixel. Then, the searching range can be narrowed from 0 to 1 and the corresponding iteration number is reduced to [0, N] and N is the number of unique values of displacements with the value from 0 to 1. According to this, a large number of the useless pixel values are excluded and speed can be enhanced greatly. 

In order to reduce the influence of large range of displacement on the calculation of objective function, quite a lot of pixels with displacements exceeding 1 pixel have to be normalized for the whole image. Generally, pixels with larger displacements and great variations in magnitudes of pixels’ movements must be classified as change class; therefore, normalization is executed only for the pixels in changed class whose displacements are more than 1 pixel. To be more elaborate, the partial normalization dynamically changes with each iteration. For ith iteration, the threshold is $w_{i}$ and pixels with displacements’ values $w_{1},\ldots ,\;w_{i}$ are automatically labeled as unchanged class $C_{1}$ and other pixels with displacements $w_{i+1},\ldots ,\;w_{n}$ which are larger than $w_{i}\;$ are classed as changed part $C_{2}$. The pixels in $C_{2}$ whose displacements $w_{j},\ldots ,w_{n}$ (j≥i+1), are greater than 1 need to be normalized to [$w_{i+1},\;w_{end}$] but the corresponding percentages remain unchanged. The formulas are as follows:

(12) $$k_{j}=\frac{w_{end}-w_{i+1}}{w_{max}-w_{min}}$$

(13) $$w_{j}=w_{end}+k_{j}\left(w_{j}-w_{min}\right)$$

$w_{end}$ is the maximum displacement value which is most close to 1; $w_{max}$, $w_{min}$ are the maximum and minimum pixel value of changed class $C_{2}$ which need to be normalized. 

Based on the two strategies, the threshold calculation can be more efficient and lay a foundation for the selection of the optimal threshold.

### 3.3. The Proposed CD Method

**Figure 5:**
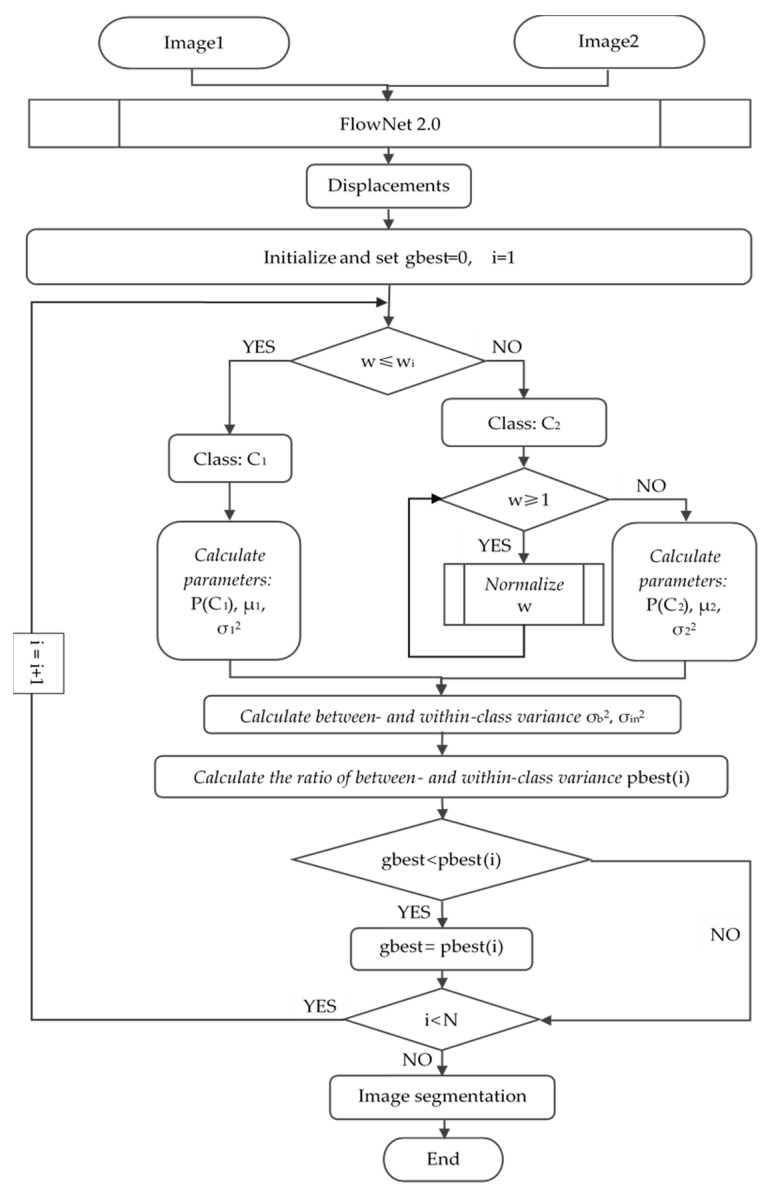
Flowchart for the proposed OFATS.

The whole flowchart of proposed OFATS is as Figure 5 shown and the details of change detection process are implemented in Algorithm 1. The essential steps of OFATS are motion detection based on FlowNet 2.0 and segmentation based on adaptive threshold selection criteria. For motion detection, the selected frames are input into FlowNet 2.0 to compute the magnitude in horizontal and vertical directions, based on which the displacements are calculated by Equation (1). After that, the next steps are the iterative process for optimal threshold selection. Following Algorithm 1, Equations (2)–(13) are repeatedly applied to calculate the fitness value for a fixed number to enable the iterative optimization. Based on the optimal threshold, the displacement result can be segmented into changed and unchanged parts.

The details of OFATS are implemented in Algorithm 1:

**Algorithm 1.** The proposed OFATS for change detection in natural disaster**Input:** The two frames extracted from the video sequence.**Output:** The change detection result. 1:   Input the two frame images and calculate the movement in horizontal and vertical directions based on FlowNet 2.0;2:   Calculate the displacements reserving a decimal fraction based on Equation (1);
3:   Generate initial global fitness value *gbest* and iteration value *i*;
4:   **while** the algorithm does not reach the termination condition **do**5:   I = I + 1; 6:   Divide into unchanged class *C_1_* and changed class *C_2_* threshold *w_i_* and normalize displacements which are larger than 1 in *C_2_* according to Equation (12) and (13) and then involve in arithmetic by using Equations (5)–(10);7:   Calculate between- and within-class variance by using Equations (3) and (4);8:   Calculate fitness value by using Equation (2);9:   **if** The solution is better **then**
10:     Replace the current individual;11:   **else**
12:     The individual does not change;13:   **End if**
14:   Find out the current global best agent;15: end while16: **return** The optimal threshold.17: Divide the image into two parts by optimal threshold value by using Equation (11). 

## 4. Data and Experiments

In this section, the proposed OFATS is applied to the detection of motion in two real video datasets including tsunami dataset and landslide dataset. The experimental results can be divided into two parts. Firstly, we verify the performance of the proposed OFATS method using video frame images with different input parameters. Secondly, the proposed method is compared with state-of-the-art CD methods using the two datasets.

### 4.1. Study Datasets

**Figure 6:**
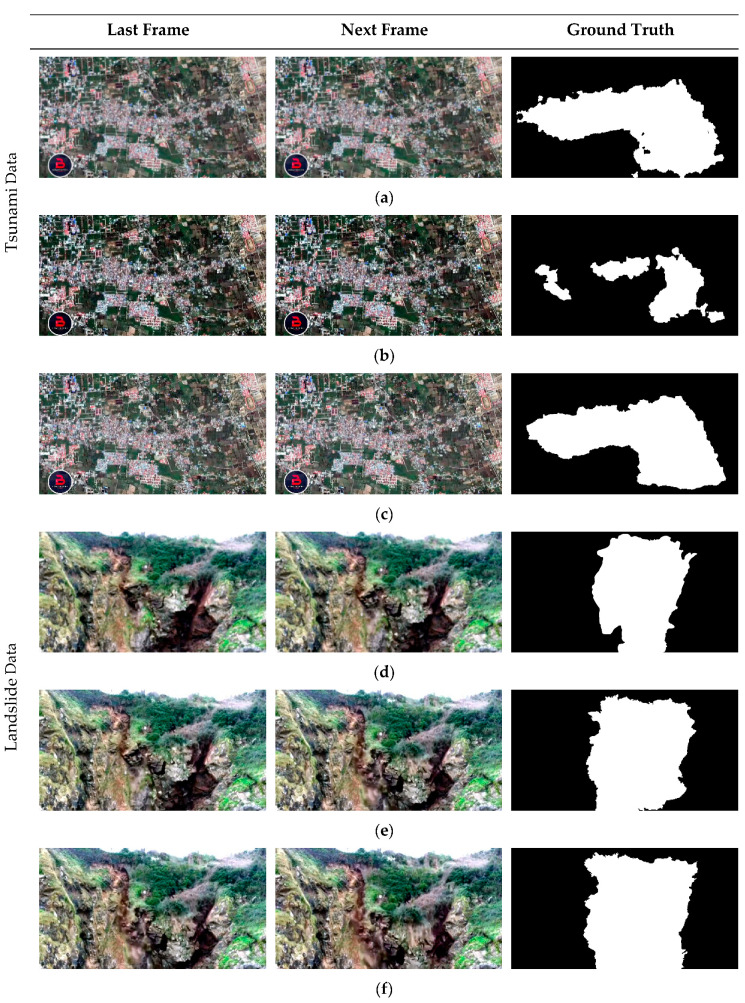
Experimental video frames and the corresponding ground truth: (**a**–**c**) Frame 160 and 165, Frame 162 and 163, and Frame 175 and 180 of tsunami video, respectively; (**d**–**f**) Frame 4960 and 4970, Frame 4970 and 4980, and Frame 4980 and 4985 of landslide video, respectively.

The proposed CD method is evaluated using two video frame datasets representing different natural disasters. The first video data gives a glimpse at tsunami in Petobo, Indonesia, where a 7.5 magnitude earthquake trigged a tsunami on 28 September 2018. Digital Globe’s WorldView captured these change progress by satellite images and transformed into a video consisting of 301 effective video clips [57]. Another example is about the slow-moving landslide produced by a subject named massive landslides caught on camera 2 and a video clip with 172 effective frames is selected [58]. In this research, we both select six frames of two video datasets but the quantities of alternate frame are different (at frame 160 and 165, 162 and 163, and 175 and 180 for the tsunami scene and at frame 4960 and 4970, frame 4970 and 4980, frame 4980 and 4985 for landslide scene, respectively) as the input image sequences for change detection in order to test OFATS’s robust to arbitrary movements. The experimental data together with the ground truth generated by visual interpretation are shown in Figure 6.

### 4.2. Evaluation of the Proposed Threshold Selection Method

In this section, the aim is to verify whether the proposed algorithm is able to automatically determine the optimal threshold for CD. Considering the change detection as a binary classification problem, the F1-measure is often used to test the selection of the optimal threshold. F1, which can synthetically consider precision (P) and recall (R) in binary classification, is shown in Table 1. The threshold that has the highest F1 will be considered as the optimum threshold. The value of F1 indicates the accuracy of change detection, and the closer to 1 means more accurate. This verification is executed on two sides: the correspondence of the optimal threshold and the maximum F1-measure value, and the performance to determine the optimal threshold value based on adaptive threshold selection proposed in OFATS and Otsu, a classic thresholding way for binarization in image processing.

**Figure 7:**
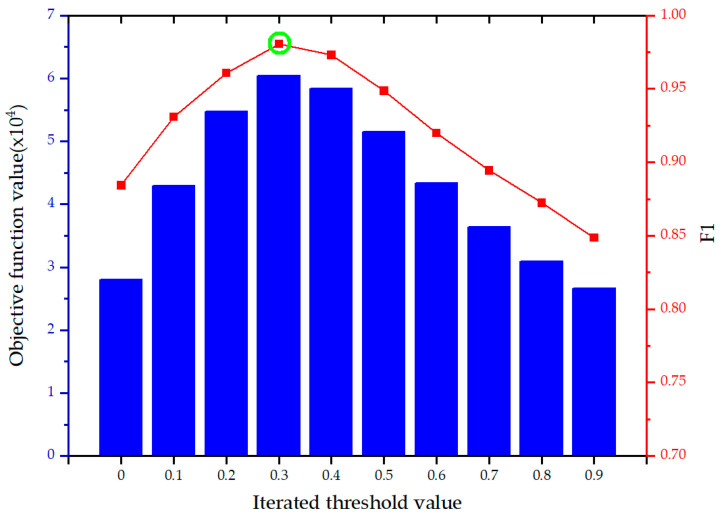
The objective function and F1 values with the variation of iterated threshold values.

In the first experiment, the frames 160 and 165 in the tsunami video are taken as an example to test whether the proposed OFATS can generate the optimal threshold with the corresponding maximum F1-measure value. If the threshold determined by the proposed objective function is in accordance with the peak value of F1, it means that the generated threshold is optimum and OFATS has the best performance. The variations of objective function value with respect to threshold value are demonstrated in Figure 7, as well as the corresponding F1 value. The blue bars represent the objective function values and the red line represents F1 values with iterated threshold values. The optimal threshold based on OFATS and the corresponding F1 are labeled with green circle. According to Figure 7, the maximum objective function value (6.04 × 10^4^) corresponds to the optimum threshold (0.3) based on which can achieve the highest peak of F1 during the whole iterations. This indicates that OFATS can automated produce the optimum threshold with the highest F1-measure value.

In order to illustrate the robustness of adaptive threshold selection in OFATS, Otsu is introduced as a comparison of the optimal threshold selection based on the same displacements’ results in the second experiment. There are three groups of tsunami data and three groups of landslide data and the comparison results based on different threshold selection methods for experimental data have been shown in Table 2. 

According to Table 2, the corresponding F1 values based on the adaptive threshold selection in OFATS are higher than Otsu in most cases, except the case in frames 4960–4970, which OFATS has the same threshold with Otsu. For the majority experimental frames, average F1 values based on OFATS are 0.02 higher than Otsu. This comparison indicates that the proposed OFATS is more robust to generate the optimum threshold thus accurately detecting the natural disaster change between video sequences.

### 4.3. Comparing the Proposed Method with Other CD Methods

This section takes frames 160–165 of the tsunami video and frames 4960–4970 of the landslide video as examples and compares the proposed algorithm with state-of-the-art CD algorithms, including Image Differencing, Image Rationing, Change Vector Analysis (CVA), Post-classification comparison (PCC), Kullback–Leibler divergence (KL), and Classic optical flow (COF) based on HS, a traditional optical flow estimation method. The aim is to test the superiority but to verify the robustness of OFATS to different range of movements.

The evaluation methods in this experiment are Producer’s Accuracy (PA), and User’s Accuracy (UA), Overall Accuracy (OA), Kappa coefficient (K). PA and UA are local indexes, where PA is obtained by dividing the number of correctly classified pixels in each class by the number of ground truth pixels in the corresponding class and UA is obtained by dividing the number of the total correctly classified pixels in the same class. Thus, there are four related indexes, that are $PA_{c}$, $PA_{un}$, PA for changes and unchanges, and $\;UA_{c}$, $UA_{un}$, UA for changes and unchanges, as shown by Equations (14)–(17). OA and K are global indexes, where OA is the proportion of number of correctly identified pixels, both changed and unchanged, to the number of total pixels, and K builds on OA by taking into account both the omission and commission of pixels. As OA, K, and F1 increase and approach 100%, 1, and 1, respectively, so too does the accuracy of the CD method in differentiating changes from non-changes.

(14) $$PA_{c}=\frac{tp}{tp+fn}$$

(15) $$PA_{un}=\frac{fp}{fp+tn}$$

(16) $$UA_{c}=\frac{tp}{tp+fp}$$

(17) $$UA_{un}=\frac{fn}{fn+tn}$$

OA and K are calculated by Equations (17) and (18):

(18) $$\mathrm{OA}=\frac{tp+tn}{tp+tn+fp+fn}$$

(19) $$k=\frac{k_{1}-k_{2}}{1-k_{2}}$$

where $k_{1}$ and $k_{2}$ are computed as follows:

(20) $$k_{1}=\frac{tp+tn}{tp+tn+fp+fn}$$

(21) $$k_{2}=\frac{\left(tp+fn\right)*\left(tp+fp\right)+\left(fp+tn\right)*\left(fn+tn\right)}{{\left(tp+tn+fp+fn\right)}^{2}}$$

**Figure 8:**
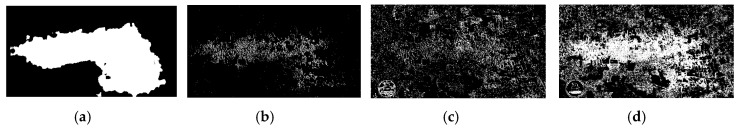

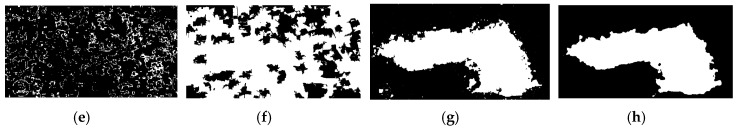
The change detection results for tsunami dataset: (**a**) Ground Truth; (**b**) Image differencing; (**c**) Image rationing; (**d**) CVA; (**e**) PCC; (**f**) KL; (**g**) COF and (**h**) OFATS.

**Figure 9:**
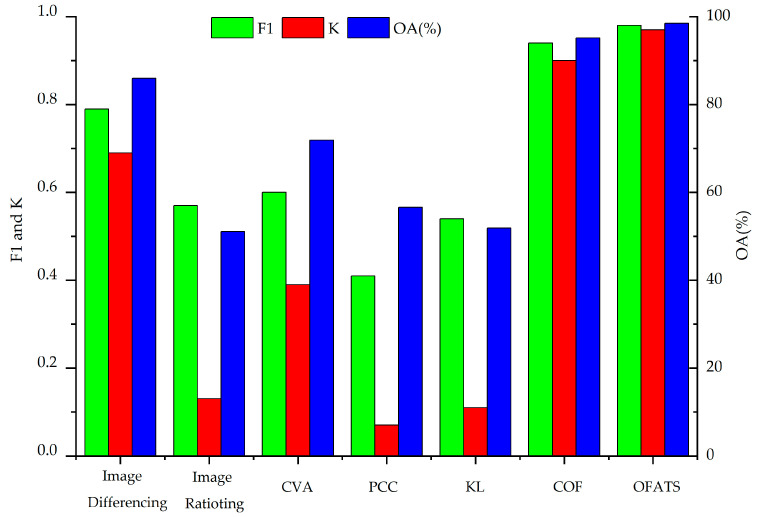
Comparison between other change detection (CD) methods and OFATS using Tsunami images.

**Figure 10:**
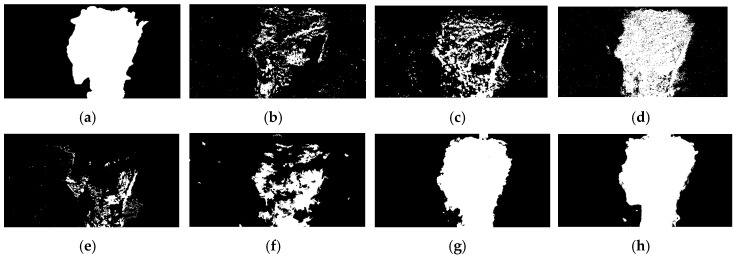
The change detection results for landslide dataset: (**a**) Ground Truth; (**b**) Image differencing; (**c**) Image rationing; (**d**) CVA; (**e**) PCC; (**f**) KL; (**g**) COF and (**h**) OFATS.

**Figure 11:**
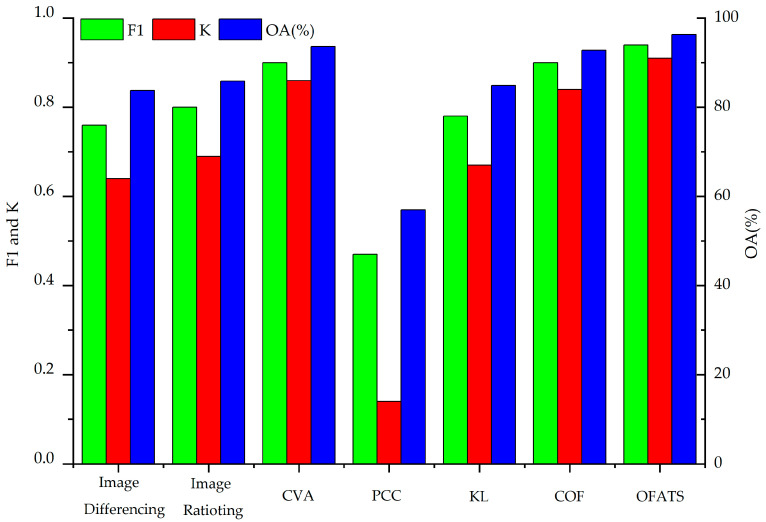
Comparison between other CD methods and OFATS using Landslide images.

All of the previously mentioned CD methods were used to analyze tsunami and landslide data, and CD maps are shown in Figures 8 and 10 and the accuracy results were tabulated in Table 3 and Table 4, respectively, and demonstrated in Figures 9 and 11, respectively. The results indicate that the proposed OFATS method has K and F1 values closing to 1 and also has the highest OA values, which shows that it is capable of accurately distinguishing between changed and unchanged pixels. The values of K, F1, and OA, according to Table 3 and Table 4, are 0.98%, 0.97% and 98.5%, respectively, for the tsunami dataset, and 0.94%, 0.91%, and 96.3%, respectively, for the landside dataset.

Figure 8a is the ground truth in which white represents changes and black means unchanges. Figure 8b–g are the results from comparative CD methods, from which most of them have difficulty to provide a clear boundary and complete changed area, especially for the CD methods of PCC (Figure 8e) and KL (Figure 8f). Many changed areas are wrongly detected as non-changed areas of image differencing (Figure 8b); however, the results of image rationing and CVA (Figure 8c,d) are the opposite. Figure 8g from COF is better than the traditional CD methods, and the result is very similar to OFATS (Figure 8h); however, the change detection accuracy of COF is less than the proposed method with several tiny false alarm areas.

To further analyze the experimental results, Table 3 presents the quantitative performance indexes of these different CD methods for the tsunami dataset and Figure 9 visually displays this. For image rationing, PCC and KL CD methods, the F1 values are around 0.5, OA is less than 60% and K is smaller than 0.15, which shows these methods fail to identify the changed area. Image differencing and CVA are slightly better than them but the results are still unsatisfying. This is because most of these CD methods only use one band of the RGB images to directly do algebraic operation or some transformation computing, which is unable to digging deep features to detect the change information of the corresponding pixel with small changes. Thus, some originally changed land cover types are hard to be tested. Moreover, the CD results are extremely fragmented and have to be implemented by morphologic erosion and dilation. The selection of the optimum threshold for the final binary image also produces errors. KL detects changes based on the spectral similarity of two single band, but the changed pixels in our research are only with displacements and no obvious spectral change in appearance. Thus, KL is less sensitive to this kind of changes that occurring when the displacements are small. PCC concurrently obtains the changed boundary and the “from-to” change information; however, PCC is the least accurate of the algorithms that were studied in this paper. Only small displacements or slight deformations occur rather than land cover type changes, resulting in PCC’s ineffectiveness in the experimental data. Both COF and the proposed OFATS method produce reasonable CD accuracy with values of F1, OA and K higher than 0.9. Compared to COF, the proposed OFATS can achieve higher accuracy because high-level features can be extracted based on deep learning and the corresponding motion results can keep a higher precision [59]. Thus, the final CD accuracy based on OFATS are higher than that based on COF even if they use the same optimal threshold value.

Figure 10 shows the results for landslide dataset. Table 4 and Figure 11 present the quantitative analysis indexes of different CD methods. By observing the CD results in Figure 10, we can still find that the proposed OFATS is the most similar one to the change ground truth.

Similar to Table 3, Table 4 also demonstrates the superior performance of OFATS with the highest F1, OA and K indexes, reaching 0.94, 96.3% and 0.91, respectively. Figure 11 also visually displayed the comparison of different CD algorithms, where the three accuracy indexes of OFATS are all the maximum but the corresponding values of PCC are minimal. The similar accuracies of the two datasets show that the proposed OFATS method maintains excellent performance and is robust to different motion changes. It should be noticed that nearly all CD methods perform better than in the Indonesia tsunami case.

**Figure 12:**
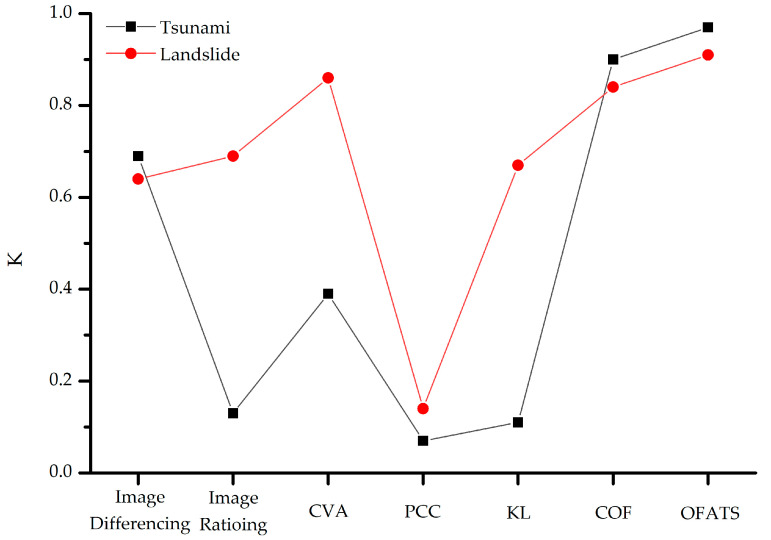
The comparison of K for experimental datasets based on CD methods.

Although the two experimental datasets are both from natural disaster scenes, the mean pixel displacement in the landslide dataset is larger than that of the tsunami dataset. The difference can be used to test the robustness of OFATS. The comparative analysis will be based on K values for all of the algorithms for both the landslide and tsunami datasets, as shown in Figure 12. The K values based on CD methods for the two datasets have varied but the change trends are basically identical. The K values for the traditional CD methods, other than image differencing, all follow the trend of the value obtained from the landslide dataset being higher than that of the tsunami dataset. It is worthwhile to be mentioned that the K values obtained from the landslide dataset are significantly higher than that of the tsunami dataset for image rationing, CVA and KL. Specially, the K values are greater than 0.6 and less than 0.4 for the landslide and tsunami datasets, respectively, which illustrates that these CD methods should only be fit for large displacements. The different performances of these CD methods indicate that only when the differences between corresponding pixels on bi-temporal images are large, like in the case of landslides, can these CD algorithms detect the change. The K values that were obtained using PCC are less than 0.2, therefore these results further indicate that PCC is not applicable for these two types of situations regardless of the magnitude of displacement. 

Despite the variations of K values, the two CD methods based on optical flow estimation algorithms achieved excellent results for both experimental datasets and K values were all greater than 0.8. However, the K values obtained using OFATS are 10% higher than COF for both datasets and their absolute values are both greater than 0.9. The superiority of OFATS is not only the accuracy, but it is also significantly more efficient in terms of computing time. Therefore, our proposed OFATS is more practical in these actual circumstances.

## 5. Conclusions

The challenging problems in natural disaster detection are how to detect the motion change and how to determine an adaptive threshold that can automatically and rapidly produce accurate change detection results. To solve this problem, an automatic change framework, termed as OFATS, is proposed in this paper. First, the displacement was computed from two frames using optical flow estimation algorithm based on deep learning. Then, the optimal threshold for rapidly separating changed from unchanged parts was automatically generated using an adaptive threshold selection based on a new objective function by narrowing the threshold searching range and dynamic normalization. 

The proposed OFATS has been applied to two different natural disaster videos. The CD results have been compared with seven other state-of-the-art CD methods, visually and quantitatively. The quantitative evaluation demonstrated that the accuracies of proposed method are greater than 95% for the two experimental datasets and it surpasses the most excellent CD algorithms by almost 4% for tsunami data and 5% for landslide data. Experiments showed three advantages of the proposed method: (1) it can detect the change using video datasets for natural disasters in an automatic way, which have rarely been studied before; (2) it is highly efficient to conduct natural disaster change detection, even for small motion; (3) it can automatically generate the optimum threshold for the following image segmentation. 

## Figures and Tables

**Table 1:** Formulas related to calculating F1.

Parameter Name	Formula	Explanation of Abbreviations
P	$\frac{tp}{tp+fp}$	tp (true positive): detects that are correctly identified as changed tn (true negative): detects that are correctly identified as unchanged fp (false positive): detects that are falsely identified as changed fn (false negative): detects that are falsely identified as unchanged
R	$\frac{tp}{tp+fn}$	
F1	$\frac{2*P*R}{P+R}$	

**Table 2:** The optimum thresholds and the corresponding F1 values based on adaptive threshold selection in OFATS and Otsu for the experimental data.

		Adaptive Threshold Selection in OFATS		Threshold Selection Based on Otsu	
Study Data	Experimental Frame	Optimum Threshold	F1	Optimum Threshold	F1
Tsunami data	Frame 160–165	0.3	0.98	0.4	0.97
	Frame 162–163	0.2	0.97	0.38	0.90
	Frame 175–180	0.3	0.99	0.48	0.96
Landslide data	Frame 4960–4970	0.4	0.94	0.4	0.94
	Frame 4970–4980	0.3	0.92	0.5	0.91
	Frame 4980–4985	0.3	0.92	0.5	0.91

**Table 3:** Confusion matrices along with indexes of the tsunami data.

Method		Ground Truth			F1	K	OA (%)
		C	U	UA (%)			
Image differencing	C	507,934	37,899	93.0	0.79	0.69	86.0
	U	237,043	1,183,204	83.3			
	PA (%)	68.2	96.7				
Image rationing	C	624,451	841,520	42.6	0.57	0.13	51.1
	U	120,526	379,583	76.0			
	PA (%)	83.8	31.1				
CVA	C	420,863	229,199	64.7	0.60	0.39	71.9
	U	324,114	991,904	75.4			
	PA (%)	56.5	81.2				
PCC	C	294,325	401,789	42.2	0.41	0.07	56.6
	U	450,652	819,314	64.5			
	PA (%)	39.5	67.1				
KL	C	548,695	750,222	42.2	0.54	0.11	51.9
	U	196,282	470,881	70.6			
	PA (%)	73.7	38.6				
COF	C	688,338	36,873	94.9	0.94	0.90	95.2
	U	56,639	1,184,230	95.4			
	PA (%)	92.4	97.0				
OFATS	C	737,237	21,164	97.2	0.98	0.97	98.5
	U	7740	1,199,939	99.3			
	PA (%)	98.9	98.2				

**Table 4:** Confusion matrices along with indexes of landslide data.

Method		Ground Truth			F1	K	OA (%)
		C	U	UA (%)			
Image differencing	C	512,244	218,623	70.1	0.76	0.64	83.8
	U	100,309	1,134,904	91.9			
	PA (%)	83.6	83.8				
Image rationing	C	540,840	205,471	72.5	0.80	0.69	85.9
	U	71,713	1,148,056	94.1			
	PA (%)	88.3	84.8				
CVA	C	577,780	90,087	86.5	0.90	0.86	93.6
	U	34,773	1,263,440	97.3			
	PA (%)	94.3	93.3				
PCC	C	380,536	613,376	38.3	0.47	0.14	57.0
	U	232,017	740,151	76.1			
	PA (%)	62.1	54.7				
KL	C	525,254	208,691	71.6	0.78	0.67	84.9
	U	87,299	1,144,836	92.9			
	PA (%)	85.7	84.6				
COF	C	611,643	140,762	81.3	0.90	0.84	92.8
	U	910	1,212,765	99.9			
	PA (%)	99.8	89.6				
OFATS	C	584,927	44,777	92.9	0.94	0.91	96.3
	U	27,626	1,308,750	97.9			
	PA (%)	95.4	96.7				
